# Complete mitochondrial genome of the marine mysid *Neomysis awatschensis* (Mysida, Mysidae)

**DOI:** 10.1080/23802359.2019.1673238

**Published:** 2019-10-16

**Authors:** Beom-Soon Choi, Young Hwan Lee, Dae-Sik Hwang, Chae Woo Ma, Atsushi Hagiwara, Jae-Seong Lee

**Affiliations:** a Phyzen Genomics Institute, Seongnam, South Korea;; b Department of Biological Science, College of Science, Sungkyunkwan University, Suwon, South Korea;; c Research Institute of Environmental Health and Safety, EHR&C, Bucheon, South Korea;; d Department of Life Science and Biotechnology, College of Natural Science, Soonchunhyang University, Asan, South Korea;; e Institute of Integrated Science and Technology, Nagasaki University, Nagasaki, Japan;; f Organization for Marine Science and Technology, Nagasaki University, Nagasaki, Japan

**Keywords:** *Neomysis awatschensis*, mysid, mitochondrial genome

## Abstract

The complete mitochondrial genome was sequenced from the marine mysid *Neomysis awatschensis*. The sequenced total genome size was 19,135 bp. The mitochondrial genome of *N. awatschensis* contained 13 protein-coding genes (PCGs), two rRNAs, and 22 tRNAs. Of 13 PCGs, all the genes had complete stop codons TAA and TAG, respectively, while the start codon of 13 PCGs was ATG (*CO1, Cytb*, *ND4L, ATP8*, *ATP6*, and *ND4* genes), ATT (*CO3*, *ND2*, and *ND5* genes), and ATA (*CO2, ND3, ND6,* and *ND1* genes), respectively. The ratio of A + T and G + C nucleotides of 13 PCGs of *N. awatschensis* mitogenome showed 68.8% and 31.2%, respectively, while those ratio of all the sequences were 70.8% and 29.2%, respectively.

To date, in the genus *Neomysis*, 18 species have been retrieved (https://en.wikipedia.org/wiki/Neomysis). Of them, complete mitochondrial genomes have been reported from *Neomysis orientalis* (Shen et al. 2015) and *Neomysis japonica* (Song et al. 2016). After the establishment of marine mysid *Neomysis awatschensis* as a standard marine toxicity test organism in China (Yan et al. 2003), their transcriptome (22,141 candidate coding contigs) was identified by RNA-seq analysis (Kim et al. 2016), while environmental toxicity testings using mysids have been attempted in response to microcystin-LR (Min et al. 2018), heavy metals (Haque et al. 2018), and insecticides (Hano et al. 2019). However, their life barcode and complete mitochondrial genome have been poorly identified, despite the increase in their importance in marine environmental ecotoxicological studies. Based on its wide global distribution in the temperate regions and ecotoxicological importance of the genus *Neomysis*, the identification of the complete mitochondrial genome will be useful to confirm their life barcode and applications for ecotoxicologial and ecophysiological studies in response to emerging pollutants.

The marine mysid *N. awatschensis* was obtained from the laboratory culture, originated from the sample mysid on May 6, 2019 from the estuarine zone of the Sihwa seawall (37°17′25.6″N, 126°34′59.0″E) by Dr. Dae-Sik Hwang and maintained in Sungkyunkwan University in South Korea. The specimen was deposited in the Biological Resources Bank of National Institute of Biological Resources under the accession no. NIBRIV0000085016. We sequenced 300 bp paired-end (PE) library of *N. awatschensis* from the whole body genomic DNA using the Illumina HiSeq 2500 platform (GenomeAnalyzer, Illumina, San Diego, CA). After *de novo* assembly was conducted by spades v3.6.0 (http://cab.spbu.ru/software/spades/) with K-mer auto, we obtained 1,363,171 contigs (N50 = 1010 bp) from *N. awatschensis*. Using CLC_overlap_reads v4.3.0.114910 (http://www.clcbio.com), joined sequences (average length 493 bp) were obtained connecting 300 bp PE sequences, mapped to contigs with clc_ref_assemble v4.3.0.114910 (http://www.clcbio.com) with gap closing and extension of both ends. Then, a single supercontig was mapped to the mitochondrial DNA of *N. awatschensis*. The total length of the complete mitochondrial genome of *N. awatschensis* was 19,135 bp (GenBank accession no. MN274520).

The mitochondrial genome of *N. awatschensis* contained 13 PCGs, two rRNAs, and 22 tRNAs. The direction of 13 PCGs of *N. awatschensis* was mostly identical to those of other *Neomysis* sister species, but the placement of *12S rRNA* and the control region were opposite (Figure 1). The ratio of A + T and G + C nucleotides of 13 PCGs of *N. awatschensis* mitogenome showed 68.8% and 31.2%, respectively, while those ratio of all the sequences were 70.8% and 29.2%, respectively.

**Figure 1.**
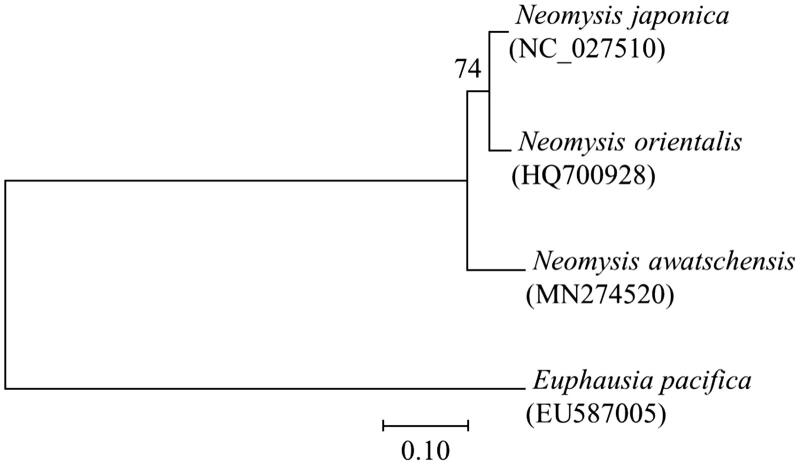
Phylogenetic analysis of mitochondrial DNA. Amino acids of 13 PCGs gene from three mysids were aligned using MEGA software (ver. 10.0.1) with the ClustalW alignment algorithm. To establish the best-fit substitution model for phylogenetic analysis, the model with the lowest Bayesian Information Criterion (BIC) and Akaike Information Criterion (AIC) scores were estimated using a maximum likelihood (ML) analysis. According to the results of model test, maximum likelihood phylogenetic analyses were performed with the LG + G + I model.

The phylogenetic placement of *N. awatschensis* was identified with the comparison of complete mitogenomes in the genus *Neomysis* (Figure 1), indicating that *N. awatschensis* was a basal of the clade genus *Neomysis*. This information will be helpful for a better understanding of mitogenome evolution in the genus *Neomysis.*

## References

[CIT0001] Hano T, Ito K, Ohkubo N, Sakaji H, Watanabe A, Takashima K, Sato T, Sugaya T, Matsuki K, Onduka T, et al. 2019 Occurrence of neonicotinoids and fipronil in estuaries and their potential risks to aquatic invertebrates. Environ Pollut. 252(Pt A):205–215. 31151059 10.1016/j.envpol.2019.05.067

[CIT0002] Haque MN, Lee DH, Kim BM, Nam SE, Rhee JS 2018 Dose- and age-specific antioxidant responses of the mysid crustacean *Neomysis awatschensis* to metal exposure. Aquat Toxicol. 201:21–30. 29859404 10.1016/j.aquatox.2018.05.023

[CIT0003] Kim HS, Hwang DS, Lee BY, Park JC, Lee YH, Lee JS 2016 *De novo* assembly and annotation of the marine mysid (*Neomysis awatschensis*) transcriptome. Mar Genomics. 28:41–43. 27189440 10.1016/j.margen.2016.05.001

[CIT0004] Min BH, Ravikumar Y, Lee DH, Choi KS, Kim BM, Rhee JS 2018 Age-dependent antioxidant responses to the bioconcentration of microcystin-LR in the mysid crustacean, *Neomysis awatschensis*. Environ Pollut. 232:284–292. 28947316 10.1016/j.envpol.2017.09.050

[CIT0005] Shen X, Sun MA, Tian M, Zhao FQ, Chu KH 2015 The first mitochondrial genome from Mysida (Crustacea: Malacostraca) reveals an unusual gene arrangement. Mito DNA. 26(2):252–254. 10.3109/19401736.2013.823185 24021004

[CIT0006] Song JH, Kim S, Shin S, Min GS 2016 The complete mitochondrial genome of the mysid shrimp, *Neomysis japonica* (Crustacea, Malacostraca, Mysida). Mito DNA A. 27:2781–2782. 10.3109/19401736.2015.1053064 26114317

[CIT0007] Yan T, Zhou MJ, Tan ZJ, Li ZY, Li J, Yu RC, Wang LP 2003 Application of *Neomysis awatschensis* as a standard marine toxicity test organism in China. J Environ Sci (China). 15(6):791–795. 14758898

